# Supplementary material for: A Novel, Quick, and Reliable Smartphone-Based Method for Serum PSA Quantification: Original Design of a Portable Microfluidic Immunosensor-Based System
Source: Cancers (Basel). 2022 Sep 16;14(18):4483. doi: 10.3390/cancers14184483 (PMC9496945; doi:10.3390/cancers14184483)
Supplement: Supplementary file 1 [file cancers-14-04483-s001.zip › cancers-1884202-supplementary.pdf]

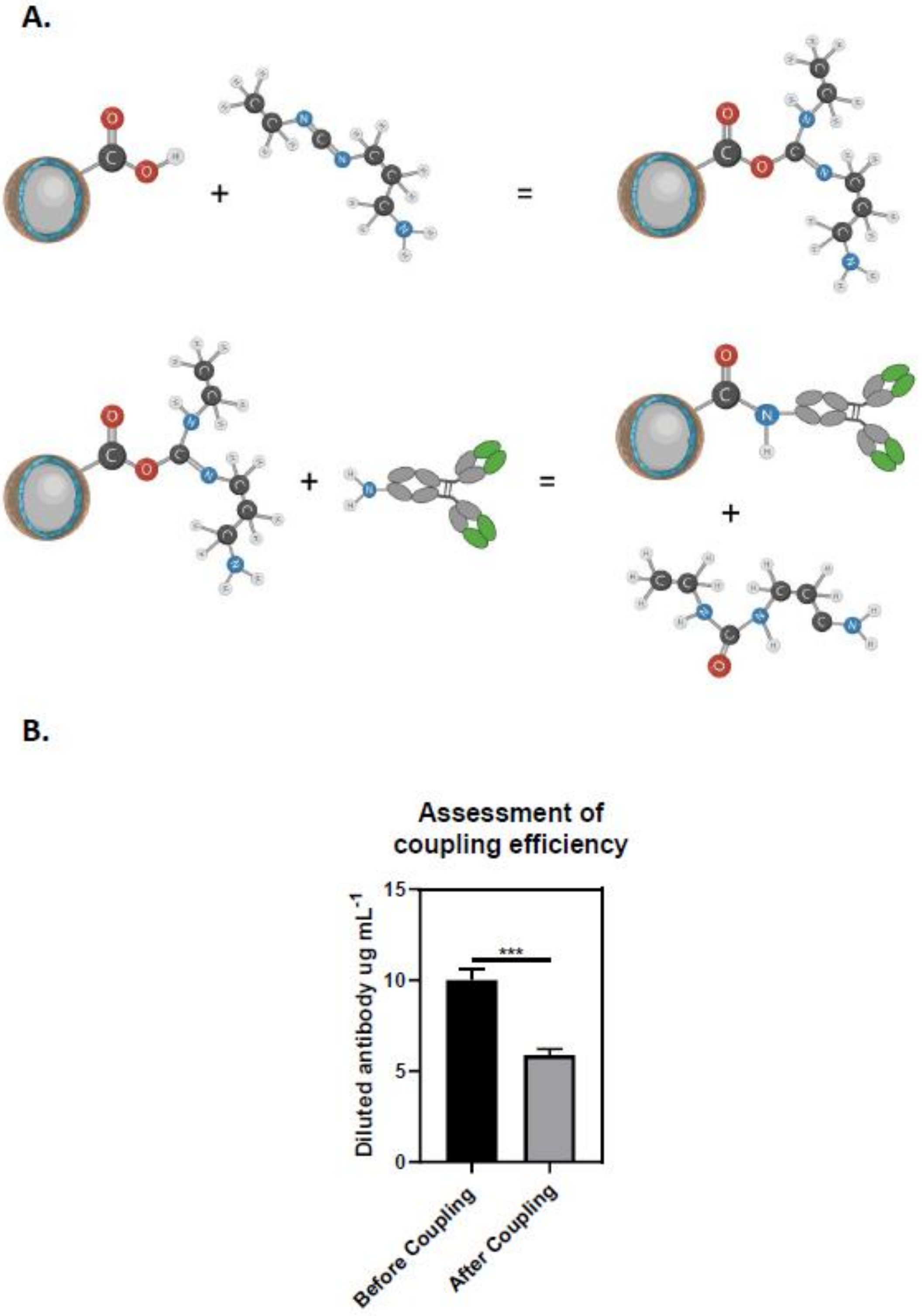

Figure S1. Schematic representation of the chemical reaction for coupling of the anti-PSA antibody to the magnetic microbeads and assessment of coupling efficiency. *** $p < 0.001$.

Table S1. Data of recruited patients

	Prostate Cancer Patients (n=50)
**Age, years. Media (range)**	66,36 (46-77)
**Nudes invasion**	24 (52,2 %)
**Seminal Invasion**	10 (20%)
**Gleason**	
≤ 6	8 (162%)
7	27 (54%)
≥ 8	15 (30%)
**Pathological Stages**	
pT2	25 (
pT3	23 (46%)

Table S2. PSA levels obtained by the proposed platform

Patient Code	Time	PSA Gold Standard (ng mL^{-1})	PSA Electrochemical (ng mL^{-1})
PR14	Basal	16,33	17,2
PR16	Basal	11,06	10,9
PR17	Basal	5,46	5,23
PR18	Basal	4,23	5,02
PR019	Basal	5,88	6,2
PR20	Basal	7,58	6,98
PR16	Basal	11,06	10,34
PR17	Basal	5,46	4,9
PR18	Basal	4,23	4,24

PR019	Basal	5,88	6,12
PR20	Basal	7,58	6,97
PR025	Basal	14,39	13,7
PR027	Basal	10,12	9,45
PR029	Basal	6,3	5,93
PR030	Basal	8,9	8,87
PR031	Basal	4,64	5,01
PR033	Basal	11,49	12,16
PR034	Basal	6,23	5,98
PR036	Basal	13	12,6
PR040	Basal	7,35	6,99
PR042	Basal	11,05	10,2
PR043	Basal	5,26	3,6
PR-AL-01	Basal	11,4	10,9
PR-AL-02	Basal	17,5	16,87
PR-AL-03	Basal	9,34	8,2
PR-AL-05	Basal	7,57	6,92
PR-AL-06	Basal	4,22	4,67
PR-AL-07	Basal	12,34	11,2
PR-AL-10	Basal	24,89	21,8
PR-AL-12	Basal	11,31	10,5
PR-AL-13	Basal	12,19	11,9
PR-AL-17	Basal	7,35	6,86
PR_AL_19	Basal	10,91	10,23
PR_AL_20	Basal	18,13	17,83

PR_AL_21	Basal	44,86	45,78
PR_AL_23	Basal	5,14	4,98
PR-AL-24	Basal	12,95	12,02
PR-AL-25	Basal	4,54	3,41
PR-AL-26	Basal	43,51	46,78
PR-AL-27	Basal	10,32	9,45
PR-AL-28	Basal	8,01	8,12
PR-AL-29	Basal	7,07	7,19
PR-AL-30	Basal	12,41	11,5
PR-AL-31	Basal	36,52	35,6
PR-AL-32	Basal	4,63	4,76
PR-AL-33	Basal	40,02	39,1
PR-JA-03	Basal	7,12	6,12
PR-JA-04	Basal	5,62	4,12
PR-JA-05	Basal	24,2	24,01
CPRC11	Basal	70,88	81,2
PR14	6 weeks	0,01	0,1
PR16	6 weeks	0,01	0,2
PR17	6 weeks	0,41	0,31
PR18	6 weeks	0	0
PR019	6 weeks	0,01	0
PR20	6 weeks	0,27	0,12
PR16	6 weeks	0,01	0
PR17	6 weeks	0,41	0,56
PR18	6 weeks	0	0

PR019	6 weeks	0,01	0,03
PR20	6 weeks	0,27	0,22
PR025	6 weeks	0,01	0,2
PR027	6 weeks	0,01	0,02
PR029	6 weeks	0	0,23
PR030	6 weeks	0,21	0,23
PR031	6 weeks	0,1	0,32
PR033	6 weeks	0,01	0,01
PR034	6 weeks	0,01	0,02
PR036	6 weeks	0,01	0,02
PR040	6 weeks	0	0
PR042	6 weeks	0,01	0,23
PR043	6 weeks	0,01	0,02
PR14	12 weeks	0,01	0,02
PR16	12 weeks	0,03	0,06
PR17	12 weeks	0,01	0,01
PR18	12 weeks	0,01	0
PR019	12 weeks	0,01	0
PR20	12 weeks	0,15	0,14
PR16	12 weeks	0,03	0,04
PR17	12 weeks	0,01	0,03
PR18	12 weeks	0,01	0,05
PR019	12 weeks	0,01	0,06
PR20	12 weeks	0,15	0,5
PR025	12 weeks	0,01	0,05

PR027	12 weeks	0,01	0,05
PR029	12 weeks	0,03	0
PR030	12 weeks	0,22	1,1
PR031	12 weeks	0,21	1,2
PR033	12 weeks	0,01	0,03
PR034	12 weeks	0,01	0,4
PR036	12 weeks	0,01	0,02
PR040	12 weeks	0,01	0,05
PR042	12 weeks	0,09	0,21
PR043	12 weeks	0,01	0,04
CPRC11-6	6 weeks	14,98	14,02
CPRC11-12	6 weeks	12,63	13,5
